# Healthy architecture map and architecture health indices in healthcare environments for mental disorders in the era of wellness revolution

**DOI:** 10.1192/j.eurpsy.2023.2098

**Published:** 2023-07-19

**Authors:** B. Abdelmoula, E. Abdelmoula, N. Bouayed Abdelmoula

**Affiliations:** 1 Genomics of Signalopathies at the service of Medicine, Medical University of Sfax, Sfax; 2 M2RCA, Ecole Doctorale Sciences et Ingénierie Architecturales (ED-SIA), Tunis, Tunisia

## Abstract

**Introduction:**

Well-being encompasses positive emotions and good physical health as well as, positive meaningful social relationships and connections or social well-being. Philosophies about the healing powers of nature, the value of spaces as a determinant of health and the impact of design of buildings on human health and wellbeing can be traced back for centuries. Bringing hospitality experience to inhospitable environments and humanizing’ healthcare environments according to a healthy architecture map and architecture health indices become currently an emergency to improve to improve the health of architectural environment and to promote the social and human wellbeing, in particular in healthcare environments.

**Objectives:**

Given the increase in mental and social health problems, We aim through this review of literature to identify as architect what is a healthy architecture map and what are the architecture health indices in healthcare environments, in particular in hospitals for mental disorders.

**Methods:**

We comprehensively review the scientific literature using Pubmed database and Google scholar to state the presence of consensual healthy architecture maps and architecture health indices in mental healthcare environments.

**Results:**

Our bibliographic review revealed that, more than for other buildings, the construction of a hospital is extremely constrained involving highly complex program and multifaceted and interconnected factors with which the architect must deal. Medical and technological progress as well as the strong involvement of the healthcare personnel, who are requested, to turn towards the patient who must be at the heart of the medical and the space process make the program more complex. This lead all professionals, to have a deep reflection on the various and multi-layered challenges of the transformation of the hospital landscape especially when it is designed for patients with mental illnesses.

**Conclusions:**

At this era of wellness revolution and precision medicine, despite some ambitious projects there are not yet a consensual healthy architecture map and precise architecture health indicators focusing on how the architectural composition of a mental health hospital may be planned.

**Disclosure of Interest:**

None Declared

